# Perception of drinking water safety and factors influencing acceptance and sustainability of a water quality intervention in rural southern India

**DOI:** 10.1186/s12889-015-1974-0

**Published:** 2015-07-30

**Authors:** Mark Rohit Francis, Guru Nagarajan, Rajiv Sarkar, Venkata Raghava Mohan, Gagandeep Kang, Vinohar Balraj

**Affiliations:** Division of Gastrointestinal Sciences, Christian Medical College, Vellore, TN 632004 India; Department of Physical Medicine and Rehabilitation, Christian Medical College, Vellore, TN 632002 India; Department of Community Health, Christian Medical College, Vellore, TN 632002 India; Society for Applied Studies, No 14, Natteri Krishnamachari Street, Krishna Nagar, Vellore, 632001 Tamilnadu India

**Keywords:** Perceptions, Unsafe water, Water quality intervention, Low income setting

## Abstract

**Background:**

Acceptance and long-term sustainability of water quality interventions are pivotal to realizing continued health benefits. However, there is limited research attempting to understand the factors that influence compliance to or adoption of such interventions.

**Methods:**

Eight focus group discussions with parents of young children - including compliant and not compliant households participating in an intervention study, and three key-informant interviews with village headmen were conducted between April and May 2014 to understand perceptions on the effects of unsafe water on health, household drinking water treatment practices, and the factors influencing acceptance and sustainability of an ongoing water quality intervention in a rural population of southern India.

**Results:**

The ability to recognize health benefits from the intervention, ease of access to water distribution centers and the willingness to pay for intervention maintenance were factors facilitating acceptance and sustainability of the water quality intervention. On the other hand, faulty perceptions on water treatment, lack of knowledge about health hazards associated with drinking unsafe water, false sense of protection from locally available water, resistance to change in taste or odor of water and a lack of support from male members of the household were important factors impeding acceptance and long term use of the intervention.

**Conclusion:**

This study highlights the need to effectively involve communities at important stages of implementation for long term success of water quality interventions. Timely research on the factors influencing uptake of water quality interventions prior to implementation will ensure greater acceptance and sustainability of such interventions in low income settings.

**Electronic supplementary material:**

The online version of this article (doi:10.1186/s12889-015-1974-0) contains supplementary material, which is available to authorized users.

## Background

It is estimated that up to 1.9 million people worldwide rely on water from ←unimproved’ sources or ‘improved’ sources that are fecally contaminated [1, 2]. Providing safe and adequate quantities of water to households has long been identified as indispensible to achieving the Millennium Development Goals (MDGs) and for progress beyond [2–4]. Recent studies have found that water from “improved” sources does not necessarily equate to “safe” water. Hence, the current estimates of the number of people having access to safe water may have been overstated [1, 2]. Populations relying on unsafe or unreliable water supplies are often exposed to water-associated pathogens, besides the additional burden of collecting and transporting water over long distances [3, 5, 6].

Of the water-associated infections, diarrhea continues to be a leading child-killer globally, despite a decrease in under-five deaths by nearly 50 % between 2000 and 2013 [7, 8]. Diarrheal infections disproportionately affect children living in Low and Middle Income Countries (LMICs). An estimated 276,000 children (nearly 38 % of all deaths attributable to diarrhea) are from India alone [9, 10]. The impact of diarrheal disease is most severe on children under two years of age, with 72 % of all diarrheal deaths occurring in this crucial stage of life [7]. Recent estimates suggest that up to 95 % of diarrheal deaths among under-five children in the high-burden countries can be averted by scaling up water, sanitation and hygiene (WASH) related interventions [11, 12].

The most effective WASH interventions in order of their estimated reductions in diarrheal risk are hand-washing, proper excreta disposal and improved water quality [5]. A 2014 systematic review and meta-analysis for LMICs revealed that even though high quality piped water offers the greatest protection against diarrheal disease, filter interventions promoting safe storage are most effective at the household [13]. While interventions aimed at improving water quality have demonstrated success in improving health and reducing diarrheal diseases among children worldwide, lack of sustained use, post-intervention, has greatly limited their benefits [14–19].

Reasons for poor adoption and lack of sustained use of water quality interventions are complex and understudied [14, 17, 20]. In rural Kenya, point-of-use chlorination with a clay pot for storage, a low-cost intervention (cost ~ USD 3) had limited success [17]. Poor adoption of the intervention appeared to be linked to cultural factors and community preferences [17]. In Vellore, southern India, a region with highly contaminated water supply, testing of low-cost water quality interventions such as solar disinfection, closed valved containers (cost ~ USD 2) and domestic chlorination (cost ~ USD 0.04 per 5 L treated water) are most effective at disinfecting or preserving the quality of disinfected water [18, 21–23]; but barriers to acceptance and sustained use prevent the targeted communities from benefiting from these interventions, placing young children at a higher risk of water-related diseases [18, 23–25].

Qualitative methods can help overcome most limitations of quantitative-only methods, and their potential in exploring complex areas such as behavior change is increasingly being recognized [26, 27]. Qualitative research has helped provide critical insight on public perceptions and preferences concerning drinking water quality in the past [28–30]. We therefore designed this study to understand parental perceptions on the effects on health of unsafe drinking water, household drinking water treatment practices and factors that influence acceptance and sustainability of an ongoing water quality intervention, with the overall aim of addressing the barriers to adoption which are known to limit the health benefits of such interventions to children and their families in rural Vellore, southern India.

## Methods

### Study setting

This qualitative study was conducted as part of a year-long intervention testing the effectiveness of a commercially available, high throughput membrane filter for providing clean drinking water among residents of three villages in Kaniyambadi block (a rural administrative unit) of Vellore, Tamil Nadu, India. Earlier studies from a number of villages in this block have documented high levels of fecal contamination in the drinking water available for public consumption, and a limited acceptability of other point-of-use methods for water treatment [18, 21, 31]. The membrane filter (Skyhydrant™ water filtration system, Skyjuice™ Foundation Inc, Australia) was a commercially available, gravity-fed, source based water filtration system that has previously been pilot-tested on the residential campus of Christian Medical College (CMC), Vellore and demonstrated potential as a low-cost solution for water disinfection in smaller communities [32]. Maintenance was accomplished through designated workers performing a weekly-backwash (with water) and a once-monthly chemical cleansing using 100 ml liquid chlorine diluted in 400 ml of water to clean the membranes, costing about INR 1,500 or USD 30 every month. Each membrane filter was placed in a designated water kiosk, *i.e.* a building (taken on lease) with a cast cement roof and there were a total of 5 kiosks in the intervention villages. Study households were encouraged to collect drinking water from the water kiosks and on average, each water kiosk supplied to 20 families every day, with up to 25 L of drinking water provided to each family for their daily use.

The three villages (two intervention villages and one control village) for the intervention study were purposefully chosen from 85 villages in the Kaniyambadi block, and all households with a child less than two years of age were invited to participate. These households were largely dependent on intermittent public water supply for drinking and other domestic use and families stored their drinking water in plastic or metal pots. Chlorination of source water was done using bleaching powder fortnightly and water was mostly consumed without further treatment [21, 23].

The intervention study commenced in October, 2013 and results of the testing of the membrane filter and the effect of providing filtered water on diarrhoea in children under-two years of age is the focus of another manuscript in preparation.

### Selection of participants for the qualitative study

Selection of participants for the focus group discussions (FGDs) was purposive and focused on parents of children participating in the intervention study described earlier. Each parent was briefed about the nature and need for the FGDs through face-to-face meetings and invited to participate by field-workers performing the diarrheal surveillance in the study villages. The meetings were held at a time and place convenient to the participants, i.e., mostly in the mornings and in a village’s community hall. Meetings were held separately with both compliant (families collecting water regularly, i.e. ≥ 3 times a week) and non-compliant (families that withdrew from the study before the study end) households in the intervention villages, and with all participating households in the control village. Three key-informant interviews were held in the villages with the elected village leaders, as they are key stakeholders who directly oversee the functioning and maintenance of all basic amenities provided to the residents of their villages.

### Methodology

This qualitative study used focus groups and key-informant interviews. Focus groups were used to capture parental attitudes, perceptions and beliefs which are not easily captured using other methods such as observations and surveys [33]. Also, focus groups allow social interactions, helping highlight differences in perceptions between participants, sometimes missed with one-to-one interviews [33]. One-to-one interviews of village leaders (key-informant’s) were also conducted to explore in-depth their attitudes and perceptions on water safety, the intervention and its sustainability in their villages.

A total of 8 FGDs were held - 3 FGDs in each of the 2 intervention villages (1 with fathers from compliant households, 1 with mothers from compliant households and 1 with either parent from non-compliant households) and 2 FGDs in the control village (1 with fathers and 1 with mothers). The FGDs and key-informant interviews were held six months after the commencement of the intervention study (between April and May, 2014). A semi-structured format was employed to collect data from the FGDs and the key-informant interviews [33]. An FGD guide containing a set of open ended questions exploring various aspects such as the effects on health of unsafe water (e.g. In your opinion, how do you think water/water quality affects the health of the child?), water sourcing for drinking and other domestic uses, water treatment in the household, feedback regarding the intervention (e.g. Can you please tell us your opinion of water provided by the machine?) and willingness to support any future plans of implementation (e.g. Will you support any future plans to establish a service for providing this water?) was developed. The FGD guide was piloted in a village that did not participate in the intervention study and modified based on feedback from the piloting. The FGD guide was also used for the key-informant interviews. All the meetings were conducted in the vernacular language (Tamil) and audio-recorded.

The interviews were conducted by a field supervisor with extensive experience in community engagement, training on interviewer ethics, probing for detail and efficient management of the discussions. The lead investigator (MRF) was present as a facilitator during the meetings and interviews, and took notes to document participant observations relevant to the qualitative analysis. Field workers were also present for the meetings. The meetings lasted between 45–60 minutes and written transcripts of responses to the various questions were maintained (in addition to the audio-recording) to validate the data, post-transcription. Any information that could identify participants was not available during transcription or retained during analysis or subsequent reporting of findings.

### Data analysis

The audio-recordings of the FGDs and the key informant interviews were transcribed verbatim from Tamil to English by a designated multi-lingual transcriber with previous health research experience. Transcripts from the audio-recording were compared to the written transcripts by the field supervisor (who conducted the FGDs and interviews) as much as possible to ensure consistency of the data transcription. Due to paucity of research on the topic of study, an inductive approach was used to identify themes from the transcripts [34]. A thematic analysis was considered appropriate to make interpretations of the collected information. Data for each of the questions were entered in spreadsheets (Microsoft Excel). Responses to the various questions were deconstructed into emerging categories and sub themes using open coding on separate spreadsheets [27, 33]. The consistency and relevance of the coding was examined through triangulation, which was conducted independently by investigators not associated with the data-collection (GN and VB). All participant quotes within the scope of this study have been presented to support the results, with additional text for clarification placed within square brackets, as necessary. Data collection and reporting of results are as per the RATS guidelines for qualitative studies (http://www.biomedcentral.com/authors/rats) and the RATS checklist for the study is available as Additional file 1.

### Ethics

The intervention study was approved by the Institutional Review Board (IRB) of CMC, Vellore; this also included ethical clearance for the qualitative study. Written informed consents were obtained from the parents of children recruited for the intervention study. This report focuses on the findings from the FGDs and key-informant interviews that involved only adults (parents of participating children and village headmen) from whom verbal consent was obtained prior to participation. No compensation was offered for participating in the meetings.

## Results

### Participants

Each FGD had between 6 – 8 participants [33], i.e. a total of 56 participants (32 mothers and 24 fathers) representing 45 households participated in the 8 FGDs. The mean (SD) age in years of respondents in the meetings was 27.7 (5.2) and 35.9 (4.5) for the mothers and fathers of compliant households respectively. Mean (SD) age in years of mothers and fathers of non-compliant households was 26.8 (3.8) and 39.5 (10.5) respectively (not shown in the analysis). All mothers were housewives; fathers were mostly skilled or unskilled laborers. Socio-demographic characteristics of the participants are presented in Table 1.

**Table 1:** Socio-demographic characteristics of the study participants

Characteristic	Groups	Number (%)
**Village**	Intervention	41 (73)
	Control	15 (27)
**Sex**	Male	24 (43)
	Female	32 (57)
**Age (in years)**	21-30	30 (54)
	31-40	19 (34)
	41-50	7 (12)
**Occupation of head of household** ^**a**^	Agriculture	4 (9)
	Skilled labor	14 (31)
	Business	3 (7)
	Service related	9 (20)
	Unskilled labor	15 (33)
**Average income (Rs. per month)** ^**a**^	<5000	7 (15)
	5000 – 7499	17 (37)
	> = 7500	22 (48)

^a^n = 46 for these categories as 10 parents were from the same household

### Health effects of unsafe water

The interviewers sought general responses for the question on the relation of water and health of young children. However, participants tended to respond with a specific disease or illness. Participants identified diarrhea or “loose stools” as the most common disease from consuming unsafe water, although responses such as vomiting, cold, fever and cough were frequent across all respondent groups and study villages. Along with diarrhea, participants also identified typhoid, cholera and malaria as the other common “waterborne or water-related” diseases: mothers in the intervention villages and fathers from the control village contributed most of these responses. It is important to note that participants across all respondent groups and villages reported diseases other than diarrheal diseases as frequently as diarrheal diseases (Table 2). Participants in the control village felt that their water was like “mineral water” and trusted it.

**Table 2:** Themes, sub-themes and issues highlighted by the participants for all questions explored

Broad theme	Sub-theme	Issues
**Health-effects of unsafe water**	Water-borne or associated^a^	Diarrhea, malaria, dengue, typhoid, cholera, elephantiasis, loose stools
	Other diseases	Vomiting, cold, fever, cough, headache, lethargy, sneezing, lack of appetite, mental retardation
**Water treatment in the household**	Conditions for treatment	Treatment only done for children, treated water for adults when sick, treating water during rainy season
	Method of treatment	Boiling, warming, filtering with a cloth or sieve
**Feedback for the study intervention**	Aesthetics of filtered water	Lesser salt in water, taste of water different, no difference in taste, better tasting water, tastes bitter like mineral water, water has better colour
	Safety of filtered water	Water appears cleaner, water doesn’t have germs or dust, filtered water is free from infection
	Health benefits of filtered water	Children falls ill less, children are healthier, savings on hospital bills
	Non-health benefits of filtered water	Food is tastier with filtered water, cooking only done using filtered water
	Other feedback	Unable to spot difference, place of provision inconvenient, no support at home to collect water
**Support for the intervention**	Complete support	Service for filtration needs to be continued, expectation of a water provision centre near respondent houses, service very useful, having intervention is added advantage to the village
	Mixed feelings	Unsure if intervention will be accepted, support from husband/wife important, Village leaders only have the power to help establish intervention, cooperation may not be possible
**Willingness to pay for clean water**	Fees willing to pay	INR 30/month (USD 0.50/month), INR 100/month (USD 1.60/month), INR 150/month (USD 2.40/ month)
	Unwilling to pay	No one will pay money for filters water, not village dwellers responsibility to pay, difficulty in collecting maintenance costs from village dwellers
	Other suggestions	Need to discuss with husband/wife, explore other ways of keeping costs down (maybe parents themselves look after maintenance and water delivery), need to pass a resolution at the village level to have such a service.

^a^Using the modified Bradley classification of water-related infections [40]

On probing why unsafe water causes these diseases, two participants said the following:

*“There are many infectious organisms in water. Children get fever, cold and cough because of these infectious organisms. If we boil and filter water, children will not be affected by disease.” (Mother, control village)*

*“We take water from home when we go out, after it finishes we buy bottled water from the shop. Drinking water bought from the shop causes fever [The respondent trusts drinking water from their village over water commercially available].” (Father, control village)*

The village headman of the control village had the following observation on the effect of unsafe water on health:

*“Generally unsafe water causes vomiting, diarrhea, malaria and typhoid. But, in our village water is safe and does not make anyone sick [The respondent feels that water in their village does not cause sickness and is safe for drinking].” (Headman, control village)*

### Water treatment practices in the household

Majority of the participants considered public water supply safe for drinking and other household use. Among those who considered public water to be unsafe for drinking, boiling drinking water prior to consumption was the most popular water purification method practiced. A few parents spoke about “warming” water before giving it to children, especially during winters. Other less popular methods employed were filtering with a cloth or sieve and filtration with ceramic filters. Participants across all categories reported filtering of water more frequently for children than for adults. This is illustrated well by the following responses:

*“We boil water only if adults are sick, otherwise we only boil water for our children.” (Father, control village)*

*“Boiled water keeps children from getting sick [The respondent feels that boiling is important to disinfect water for children].” (Headman, intervention village)*

An important response tending to represent existing deficiencies in locally practiced methods of water filtration was:

*“If we filter water, we can filter only the dirt but not germs [This respondent possibly refers to filtering with a cloth or sieve, seeming to understand that this method does not remove microbiological contamination].” (Father, intervention village)*

Another participant drew attention to the importance of water filtration during rainy-season, when water might be of poorer quality than normal:

*“We filter water only during rainy season, because water is dirtier then [The respondent means that the water appears ‘visibly’ dirtier during rainy season].” (Non compliant mother, intervention village)*

### Feedback for the study intervention

Most parents reported that the filtered water from the intervention was clean, good for drinking and that they were generally satisfied with the water. A few participants from each of the respondent groups in the intervention villages reported that the water had “less taste” or “saltiness” when compared to water available in the public domain (Table 2).

Some parents reported health benefits and savings on expenses for hospital treatment and savings in fuel for boiling. The most salient responses were:

*“Children are not affected by illness now. Earlier children had many health problems. Children are well from using this water.” (Mother, intervention village)*

*“Children are healthy now. We do not visit the hospital frequently now, earlier we had to go to the hospital very often.” (Father, intervention village)*

*“We do not need to boil water anymore, so gas is saved because of the clean water [The gas referred to here is liquid petroleum gas (LPG) cylinders used as cooking fuel].” (Mother, intervention village)*

A mother spoke of how dirty the overhead village tank water used to be before the intervention study:

*“The tank water we used to drink before this study had dust and sometimes even worms [‘Tank water’ referred to here is water that is stored in overhead tanks prior to distribution through taps].” (Mother, intervention village)*

Non-health benefits of the filtered water for cooking are captured in the following response:

*“I don’t like to cook with unfiltered water now. Cooked rice is white in color [because water is cleaner] with the water you provide, sambar (lentil soup) tastes much better too.” (Mother, intervention village)*

However, a few participants were dissatisfied with the “taste” of the water:

*“Provided water is less tasty than normal water. Salt is less in the provided water.” (Father, intervention village)*

*“The water you provide is bitter [bitter could also mean tasteless], like mineral water we get in packets, so we don’t like it.” (Non compliant mother, intervention village)*

Non compliant households tended to highlight difficulties in fetching water since they did not have a person for this chore; one respondent did not think there was any difference between the filtered water and water normally available to the village:

*“The male members in the family find it difficult to bring the water. The water in our village is the same, isn’t it? [The respondent finds it hard to understand the difference between the intervention water and water available in her village]. We are dependent on the men of the house to fetch water.” (Non compliant mother, intervention village)*

### Support for the intervention

Most parents thought that having safe drinking water was beneficial, especially for the health of their children. There was however a mixed response across the different respondent groups and study villages on the need for such a water filtration intervention. A few concerns raised by the respondents highlighting issues such as lack of household support (mainly from the mothers) and village administrative support, and poor access are listed below:

*“It is doubtful whether the women will accept such an intervention for safe drinking water [acceptance here possibly means need].” (Father, control village)*

*“The water is useful to us, but our husbands have to cooperate.” (Mother, intervention village)*

*“What can we do about it? Please consult with the Panchayat president (Headman) [The respondent expresses helplessness and feels that any decision for implementation of a safe water service must come from the village leaders].” (Mother, control village)*

*“We expect a centre near our area, the water is needed.” (Non compliant mother, intervention village)*

The Headmen in two villages asserted that such a water quality intervention was valuable for reasons such as:

*“Having a water filtration system is an added advantage to our village.” (Headman, intervention village)*

*“I want my village residents to have clean and filtered water, we need the service.” (Headman, control village)*

### Willingness to pay for clean water

The participants were also asked if they were willing to pay a small fee for the establishment of a community-run clean water service (maintenance and running costs). None of the participants reported spending any money for purchasing drinking water prior to the study, except when travelling.

Reponses from participants were mixed; while some readily agreed, some were reluctant to pay:

*“A monthly contribution of about one rupee a day (INR 30 or USD 0.50 per month) should be fine.” (Father, intervention village)*

*“If we can share the expenses, then the costs will reduce.” (Father, control village)*

*“I have to discuss with my husband to see if we can pay to support the service.” (Mother, intervention village)*

*“No one will pay for water.” (Father, intervention village)*

The headmen were unanimous in their opinion that a payment system for drinking water may be difficult to sustain in rural settings:

*“I have to talk to my people to see if they are willing to pay for a service to provide clean drinking water.” (Headman, intervention village)*

*“If we insist on them contributing towards maintenance or operation costs for water filtration, they will prefer to use unfiltered drinking water from their house connections [‘house connection’ refer to public water connection made available within the household premises for villagers who can afford it].” (Headman, intervention village)*

The difficulty in charging a fee or collecting money for such a water filtration service is best addressed by the following statement by the Headman in the control village:

*“Spending money for water is a feature of towns and cities, but in villages it is not so [people do not pay for drinking water]. In villages people are not even ready to pay the water tax [minimal fee for the provision of drinking water every month collected by the village administration]. It is therefore hard to collect money for a water filtration service.” (Headman, control village)*

## Discussion

Qualitative methods employed in this study allowed the researchers to gain insight into participant perceptions regarding the health-effects of consuming unsafe water and water treatment in practices in rural Indian households. The inductive, “bottom-up” approach used for analysis of the responses [34] helped identify key factors that influence acceptance and sustainability of water quality interventions in rural southern India. This information can be used in the future for better planning and effective implementation of such interventions in low income settings.

Parents of young children linked unsafe water not only with diarrheal disease, but also with other unrelated diseases such as cough, cold and fever. A possible reason for this could be the concept on “hot” and “cold” foods that is widely prevalent in rural Indian families [35]. Consumption of “hot” and “cold” foods (and beverages) are believed to cause illness and are avoided, especially when a person is ill. In an earlier study in the same area, participants perceived “heating” of the body to be an important cause of diarrhea; very few felt that diarrhea was caused through consumption of unclean water [23]. This lack of consistency of beliefs is an important impediment to acceptance of such interventions and highlights the need for repeated educational and behavior change interventions in these communities for maximum impact.

Boiling (a relatively expensive method [36]) cloth (an ineffective method [13]) were the commonest household water treatment, methods practiced. Boiling was resorted to only for young children and sick adults. In addition some families “warmed” water before giving it to young children, which is also related to “hot” and “cold” food concept mentioned earlier. A few parents felt that water bought while travelling (“outside” water) can cause illness, and placed greater trust on water sourced from their village. Parents from the control village compared their water to “mineral” water (generally meaning that it appeared clean and tasted good), which conferred a false sense of protection. The practice of warming (vis-à-vis boiling) and the false sense of protection from the locally available drinking water may place young children at the greatest risk of diarrheal illnesses as high levels of fecal coliform contamination have consistently been documented in public drinking water samples from urban and rural areas of Vellore region [18, 21–23]. Hence, there is a pressing need for educating local communities, especially parents of young children on making drinking water potable, one of which is boiling and safe storage [18, 21, 31].

The importance of taste and odor of drinking water has been highlighted in studies from this region and elsewhere [17, 18, 37]. A few parents reported that the water lacked taste (saltiness) while others described the water as “bitter”. The membrane filter is not known to alter the physical composition of water drastically [21], and this feedback could represent the participants’ preference for drinking water to have a taste similar to that of untreated water. Any water intervention, hence, needs to be sensitive and address this aspect.

Parents from all study villages generally thought that filtered water was useful and provided health benefits especially to their children. However, a few parents (mostly from the non-compliant families) did not recognize any health benefit and found no need for the filtered water. The control village headman’s statement below captures the need for educating such parents and the public on the risks of consuming unsafe water:

*“People may only realize the need for clean water when they experience diseases like diarrhea and vomiting after drinking unclean water.” (Headman, control village)*

The ability to recognize health benefits from interventions is crucial to their long term acceptance and sustained use.

Distance of households from the water distribution centers was identified as an important factor affecting compliance to the intervention: non-compliant families tended to reside further away from the water distribution centers than compliant families. Lack of support from the male members to fetch water was also found to negatively influence household compliance, even though the female respondents recognized the benefit of filtered water for their children. Likewise, willingness to pay for future maintenance of the membrane filter was an important decision that mothers would not make without consulting their husbands. The dynamics of decision-making in rural households of India and South Asia is under researched and studies capturing the “process” of decision-making are vital to the success of future community based interventions.

Many parents were willing to pay approximately INR 30 (USD 0.50) every month for the maintenance of the membrane filter. Few parents recognized monetary savings with regards to expenditure towards treatment of illness in children and fuel costs for boiling. Parents who recognized these savings were willing to pay to support future maintenance of the membrane filter. Even though a service fee is currently levied for households with a private connection, (which is same as that of maintaining the membrane filter) the village headmen felt that any model with a service fee will not succeed. Most services in Tamil Nadu state (health care, education, food rationing and water and sewerage) are heavily subsidized or free in the villages [38]. Therefore, residents of the study villages generally expected clean drinking water from the government at no additional cost to them.

An important limitation of the FGD is the potential for confirmation bias and ‘socially desirable replies’. Although, individuals were encouraged to express alternate viewpoints through probing questions [39], it is possible that some may only have revealed perceptions that the community believes in. The use of purposive sampling was yet another limitation: only mothers and fathers of children less than two years of age (aged 21 – 40 years) were invited to participate, thus potentially resulting in a selection bias. Inclusion of older participants would have provided a more comprehensive understanding of changing perceptions and needs across different stakeholders. Also, the findings of this study may not be representative of parental perceptions in other rural parts of India. Moreover, parents from non-compliant families were less responsive during the FGDs, and key-informant interviews may have captured their opinions better. Additionally, we could not collect information on other aspects such as storage of water in the household and participant opinions on what water was considered “safe” to drink which, if collected, may have provided a more comprehensive snapshot of existing perceptions.

## Conclusions

This study describes gaps in the knowledge of the transmission pathways of diarrheal illnesses as well as changing perceptions over time. This is the first study in the region where the parents of young children were probed on factors likely to influence acceptance and sustainability of a low-cost water quality intervention in a low income setting. Recognizing health benefits, ease of access to water distribution centers and the willingness to pay for intervention maintenance were important factors facilitating acceptance and sustainability. Faulty perceptions of water treatment and a false sense of protection from water locally available not only place young children at the greatest risk of diarrheal diseases but mitigate the demand for water quality interventions. Taste and odor have been highlighted as key factors influencing the acceptance of drinking water in this study and in earlier ones from the region; the value of this in the acceptance and sustainability of future water quality interventions cannot be overstated. A lack of support from male members of the household further impeded long term sustainability of the intervention.

Notwithstanding the identified barriers, the need to effectively involve communities at important stages of implementation is crucial to the long term success of water quality interventions. Timely research on the factors influencing uptake of water quality interventions prior to implementation will ensure greater acceptance and sustainability of such interventions in low income settings.

## Additional file

Additional file 1: RATS checklist for the study. ᅟ

## Abbreviations

MDG: Millennium Development Goals
LMIC: Low and Middle Income Countries
CMC: Christian Medical College, Vellore
FGD: Focus Group Discussion
