# Supplementary material for: Perception of drinking water safety and factors influencing acceptance and sustainability of a water quality intervention in rural southern India
Source: BMC Public Health. 2015 Jul 30;15:731. doi: 10.1186/s12889-015-1974-0 (PMC4520261; doi:10.1186/s12889-015-1974-0)
Supplement: Additional file 1: RATS checklist for the study. — ᅟ [file 12889_2015_1974_MOESM1_ESM.docx]

| **No. Item** | **Guide questions/description** | **Reported on Page #** |
| --- | --- | --- |
| **R** |  |  |
| 1 | Research question explicitly stated | Pg.4, line 23 to Pg.5 line 2 |
| 2 | Research question justified and linked to the existing knowledge base (empirical research, theory, policy) | Pg.4, lines 5 - 23 |
| **A** |  |  |
| 3 | Study design described and justified i.e., why was a particular method (e.g., interviews) chosen? | Pg.7, lines 9 - 15 |
| **T** |  |  |
| 4 | Criteria for selecting the study sample justified and explained | Pg.6, lines 21 - 22 |
| 5 | Details of how recruitment was conducted and by whom | Pg.6, lines 22-25 |
| 6 | Details of who chose not to participate and why | NA |
| 7 | Method(s) outlined and examples given (e.g., interview questions) | Pg.7, line 10 to Pg. 8, line 16 |
| 8 | Study group and setting clearly described | Pg.5, line 6 to Pg.6, line 18 |
| 9 | End of data collection justified and described | Pg.7, lines 12 -13 |
| 10 | Do the researchers occupy dual roles (clinician and researcher)? Are the ethics of this discussed? Do the researcher(s) critically examine their own influence on the formulation of the research question, data collection, and interpretation? | NA |
| 11 | Informed consent process explicitly and clearly detailed | Pg.6, lines 22 - 24 & Pg.9, lines 12 - 14 |
| 12 | Anonymity and confidentiality discussed | Pg.8, lines 16 - 17 |
| 13 | Ethics approval cited | Pg.9, lines 2 - 4 |
| **S** |  |  |
| 14 | Analytic approach described in depth and justified | Pg.8, lines 11 - 22 |
| 15 | *Indicators of quality:* Description of how themes were derived from the data (inductive or deductive)/Evidence of alternative explanations being sought/Analysis and presentation of negative or deviant cases | Pg.8, lines 17 - 21 |
| 16 | Description of the basis on which quotes were chosen | Pg.8, lines 21 - 22 |
| 17 | Method of reliability check described and justified | Pg.8, lines 12 - 16 & Pg.8, lines 20 - 23 |
| 18 | Findings presented with reference to existing theoretical and empirical literature, and how they contribute | Pg.16, line 10 to Pg.19, line 16 |
| 19 | Strengths and limitations explicitly described and discussed | Pg.16, lines 2-8 & Pg.19, lines 1 - 16 |
| 20 | Evidence of following guidelines (format, word count), Detail of methods or additional quotes contained in appendix, Written for a health sciences audience | Format - followed, Word count (excluding abstract and references): 5,500, No additional quotes |
